# Mobile health use predicts self-efficacy and self-management in adolescents with sickle cell disease

**DOI:** 10.1093/tbm/ibab041

**Published:** 2021-05-05

**Authors:** Anna M Hood, Cara Nwankwo, Ashley Walton, Emily McTate, Naomi Joffe, Charles T Quinn, Maria T Britto, James Peugh, Constance A Mara, Lori E Crosby

**Affiliations:** 1 Developmental Neurosciences, UCL Great Ormond Street Institute of Child Health, London, United Kingdom; 2 Oklahoma State University, Department of Psychology, Stillwater, OK, USA; 3 Dartmouth College, Department of Philosophy, Cognitive Science Program, Hanover, NH, USA; 4 Division of Behavioral Medicine, Cincinnati Children’s Hospital Medical Center, Cincinnati, OH, USA; 5 Department of Pediatrics, University of Cincinnati College of Medicine, Cincinnati, OH, USA; 6 Cancer and Blood Diseases Institute, Division of Hematology, Cincinnati Children’s Hospital Medical Center, Cincinnati, OH, USA; 7 Division of Adolescent and Transition Medicine, Cincinnati Children’s Hospital Medical Center, Cincinnati, OH, USA; 8 James M. Anderson Center for Health Systems Excellence, Cincinnati Children’s Hospital Medical Center, Cincinnati, OH, USA

**Keywords:** Mobile health, Self-management, Pain, Clinical trial, Mood, AYA

## Abstract

Sickle cell disease (SCD) is associated with significant health challenges that often worsen during adolescence. Living with SCD requires a substantial amount of self-management and mobile health (mHealth) holds considerable promise for assessing and changing behaviors to improve health outcomes. We integrated a mobile app as an adjunct to a group intervention (SCThrive) and hypothesized that more engagement with the mHealth app would increase self-management and self-efficacy for adolescents and young adults (AYA) with SCD. Twenty-six AYA ages 13–21 years (54% female; 46% HbSS genotype; all African-American/Black) received six weekly group sessions (three in-person, three online). Participants were provided with the mobile app (iManage for SCD) to record progress on their self-management goals and log pain and mood symptoms. The Transition Readiness Assessment Questionnaire (TRAQ-5) assessed self-management skills and the Patient Activation Measure (PAM-13) assessed self-efficacy at baseline and post-treatment. Logging on to the app more frequently was associated higher mood ratings (*r* = .54, CI[.18, .77], *p *= .006) and lower pain ratings (*r* = −.48, CI[−.77, −.02], *p* = .04). Regression analyses demonstrated that after controlling for scores at baseline, the number of logins to the app predicted self-management skills (*p* = .05, *η*^2^ = .17) and possibly self-efficacy (*p* = .08, *η*^2^ = .13). Our study findings indicate that it can be challenging to maintain engagement in mHealth for AYA with SCD, but for those who do engage, there are significant benefits related to self-management, self-efficacy, and managing pain and mood.

## Teaser

Adolescents and young adults (AYA) with sickle cell disease (SCD) experience health challenges during the move from pediatric to adult care. Self-management (i.e., prioritizing and completing goals) and self-efficacy (i.e., confidence) are needed to manage a chronic disease effectively, but AYA with SCD often lack the skills. Mobile health (mHealth) technology (e.g., smartphones and tablets) present a way for AYA to monitor and track symptoms, change behaviors, and complete goals. We included a mHealth app into a group intervention (SCThrive) to see if using components of the app more frequently increased self-management and self-efficacy for AYA with SCD aged 13–21 years. We found that logging on to the app more often was related to better mood symptoms and lower pain ratings. AYA who logged onto the app more often reported more self-management skills and self-efficacy. Our findings indicate clinical benefits for those AYA with SCD who engaged with the mobile app.

**Implications**

**Practice**: Mobile health technology can be utilized to improve self-management and self-efficacy among adolescents and young adults with sickle cell disease.

**Policy**: Policymakers who want adolescents and young adults with sickle cell disease to engage in positive health behaviors should consider the implementation of self-management mobile health tools.

**Research**: Future research should examine ways to increase engagement in mobile health tools that can be sustained and maintained over long periods.

SCD is the most common inherited blood disorder affecting more than 5 million individuals worldwide [1] and over 100,000 people in the United States [2]. SCD most often occurs in individuals of African descent. However, greater geographic mobility has resulted in the migration of substantial populations from high prevalence areas (i.e., Sub-Saharan Africa) to low prevalence areas (i.e., Europe) over the last 10–15 years [3]. In recent years, medical outcomes for children with SCD in high-income countries have gradually improved, mostly as a result of developments in treatments such as hydroxyurea (HU) [4]. Nonetheless, health challenges often worsen in adolescence when caregivers begin to transfer responsibility for disease management [5] and during the transition from pediatric to adult care [6].

Living with a chronic condition such as SCD requires the patient to have adequate skills to be an informed and active participant in their care [7]. One essential set of skills needed for AYA with SCD as they navigate complex medical systems are those of self-management [8]. Self-management of a chronic condition like SCD requires the patient to have the ability to engage in health behaviors that help to effectively manage their disease [9]. Another component essential to care is self-efficacy, which reflects confidence in the ability to exert control over one’s motivation, behavior, and social environment [10]. Good self-management and self-efficacy involve successfully navigating ongoing symptoms and treatment along with the physical and psychological consequences inherent to the disease [8,11]. Specifically for AYA with SCD, this means responding to fever or pain symptoms with medications or behavioral strategies, regularly attending clinic appointments, adhering to medications, and having self-awareness about psychological challenges [12]. Unfortunately, previous research indicates that AYA with SCD often lack the ability, confidence, and skills to manage their disease effectively [13,14].

mHealth technology is the use of mobile devices to support medical and public health practice [15]. The use of mobile technologies (e.g., smartphones, tablet computers) has become near ubiquitous [16], especially by AYA who are “technology natives” [17] and AYA with SCD have wide access to smartphones and other electronic devices [8,18]. Given these generational and population specific attributes, mHealth interventions have the potential to transform how we provide patient health care services for AYA by overcoming logistical barriers whilst fostering greater acceptance and engagement [19]. Relatedly, mHealth holds considerable promise for improving self-management and self-efficacy skills and promoting health outcomes for AYA with SCD [20]. Some mHealth interventions for AYA with SCD have demonstrated promising feasibility and acceptability; however, most studies have focused on medication adherence, are not part of a randomized control trial (RCT), or do not yet have quantitative mHealth data in relation to primary outcomes [21,22]. As such, current evidence for mHealth interventions is modest and more studies are needed to assess efficacy and effectiveness and their ability to improve self-management and self-efficacy in AYA with SCD [8].

Our previous feasibility study utilized a qualitative design and demonstrated that AYA with SCD would use mHealth technology and that it was beneficial for tracking health behaviors [23]. AYA and medical providers participated in co-creation sessions to develop strategies for addressing self-management barriers to be incorporated into a mHealth app. Using design thinking that utilizes a problem-solving approach to match needs/preferences to feasibility, the research team independently coded interviews of AYA with SCD for themes using grounded theory [24]. Through this co-design process, we developed a mobile app. Subsequently, our pilot RCT demonstrated that a self-management intervention (SCThrive) in which AYA in the treatment arm used the mobile app and allowed participants to monitor and track their pain and mood and create and complete self-management goals, resulted in clinically meaningful changes in patient activation and self-efficacy relative to an attention control condition [25]. Nineteen AYA with SCD also completed individual semistructured phone interviews after the RCT. AYA reported that the intervention was highly feasible due to the mixed in-person/online format and acceptable because they learned skills to manage SCD. Action planning and pain/mood tracking were identified as key components in motivating self-management. AYA perceived peer support and the app as highly beneficial [26].

In the present study, our goal was to evaluate engagement with the adjunct mobile app for AYA with SCD who received the intervention to determine the specific features that were efficacious. We hypothesized that AYA with SCD would regularly use the mobile app to track daily pain, fatigue, and mood symptoms and that they would create and complete individual self-management goals. Additionally, we hypothesized that more engagement with the mHealth app would result in increased self-management and self-efficacy for AYA with SCD.

## METHODS

### Participants

We analyzed data from a pilot single-site, RCT (NCT02851615) [25]. Quantitative data presented here only include participants from the treatment arm (*N* = 26) who received the intervention and used the adjunct mobile app. Qualitative feedback regarding acceptability and feasibility of the mobile app are reported elsewhere [26]. Inclusion criteria for the study were (1) age between 13 and 21 years, (2) a confirmed diagnosis of SCD (any genotype), and (3) being on or eligible for disease-modifying therapies (e.g., HU, chronic transfusions). We excluded AYA if they were non-English speakers or if they had a psychiatric disorder or cognitive impairment that would impede study participation. Before randomization, we blocked AYA on age (13–17/18–21 years) and disease severity (severe/not severe). AYA were characterized as having severe symptoms if the review of the electronic medical record (EMR) indicated three or more pain episodes in the past 3 years and a previous history of stroke or acute chest syndrome.

### Procedure

**Figure 1 |:**
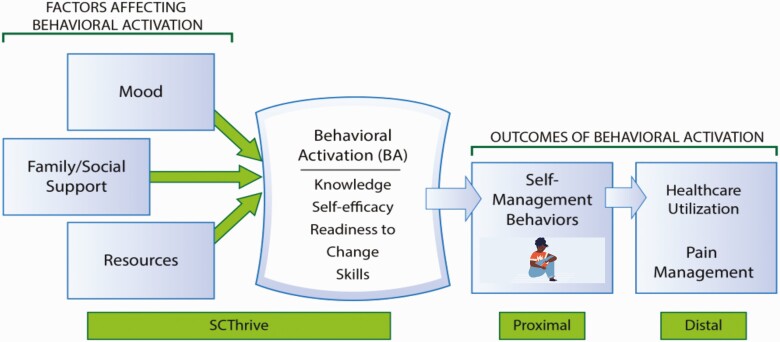
The relationship between behavioral activation, self-management, and health outcomes

A detailed description of the intervention and AYA characteristics in both treatment arms is described elsewhere [25]. Briefly, SCThrive is a multicomponent, peer-based, developmentally appropriate intervention designed to increase behavioral activation [27]. Behavioral activation is a key mechanism that impacts an individual’s likelihood for self-management and eventually impacts health outcomes [27] (see Fig. 1).

A trained clinical research coordinator identified AYA using the EMR and approached eligible participants by mail, phone, or at an SCD clinic visit. After informed consent/assent, AYA were randomized to the intervention (*N* = 27). One participant was excluded after randomization because of a cognitive issue, so 26 AYA received six weekly 90-min group sessions (three in-person, three online) guided by two facilitators (psychologists, psychology fellows, or psychology graduate students). The sessions included culturally sensitive motivational interviewing, social skills, and cognitive behavioral strategies to enhance behavioral activation. An in-person booster session was held 2 weeks after the last session. If AYA missed any online session, they could watch a recording of the missed session [25].

The study team provided AYA with the mobile app installed on an iPad and trained AYA how to use app features. AYA (or caregiver if AYA < 18 years) consented that the participant would be the only user, that they would report loss or damage immediately, and that they would log on to the mobile app from a private location. AYA were asked to return the iPad at the post-treatment assessment. Seven AYA (27% of the sample) returned the iPad after this assessment. These participants were permitted to continue using the app until the iPad was returned. AYA completed measures assessing self-management and self-efficacy at baseline and 12–14 weeks later at a post-treatment follow-up visit (4 weeks after online booster session). “Participants received $35 compensation for each completed session for a maximum of $245 (i.e., six sessions plus one booster). They received an additional $5 per session if they used the app to track their daily pain and mood symptoms and documented progress on their self-management goals.”

### Measures

#### iManage for SCD App

**Figure 2 |:**
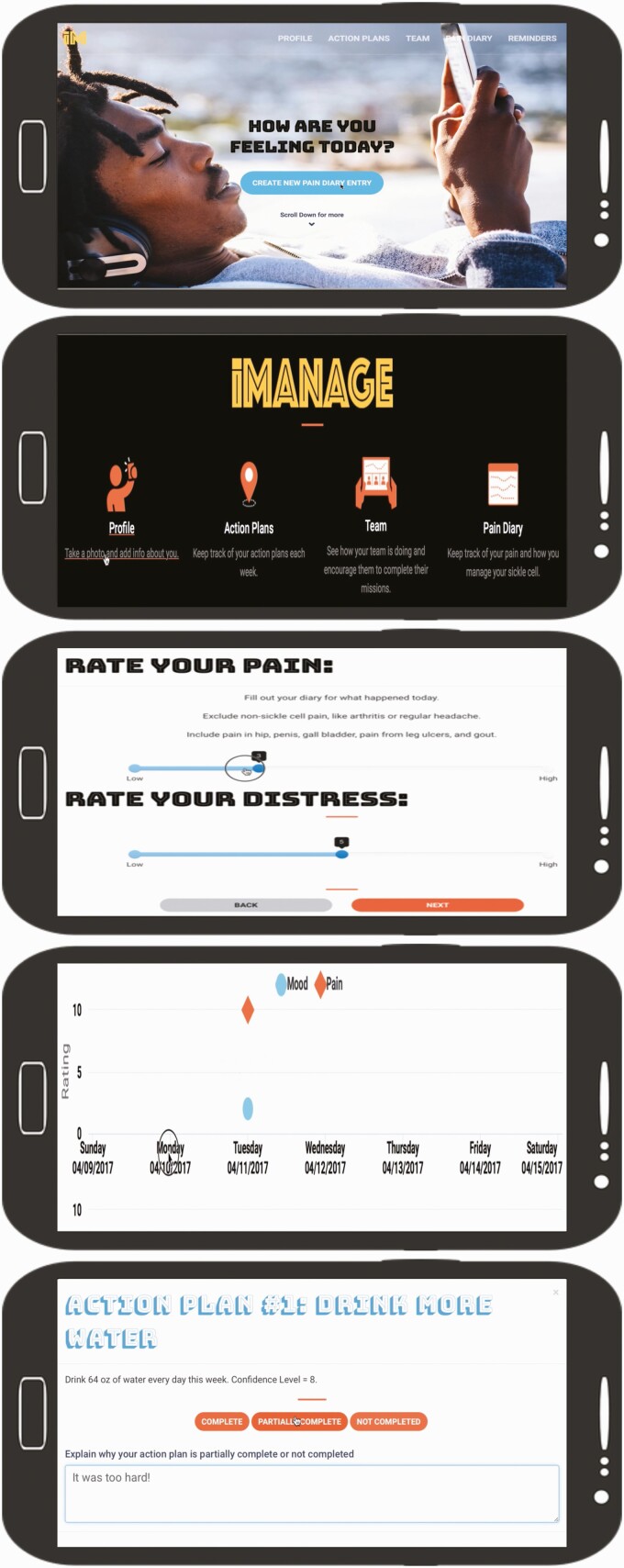
iManage for SCD App co-designed by AYA with SCD that includes customizable profile, ability to track pain and mood, set self-management goals, and track progress on goals

Before the present study, patients with SCD and medical providers helped to design the mobile app [23]. The mobile app is accessible via a smartphone or tablet on both the iOS and Android platforms. The app was written using Cordova with a.NET application programming interface (API). Data are automatically encrypted and AYA received unique login credentials. Data were backed up daily and stored on our institution’s server [28]. App users could track their daily pain and mood symptoms and create, monitor, and complete self-management goals (e.g., exercising, taking medications, sleeping). They could see the progress of other users (e.g., goals complete, not completed), but not the specific content. In addition, participants could also link SCD symptoms to their goals in a visual calendar (see Fig. 2).

We measured engagement in the mobile app by (i) dividing the number of logins by the number of days with access to the app (e.g., logins/access days) and (ii) the calculating number of completed self-management goals. AYA also rated their daily pain (1 = very low, 10 = very high) and mood (1 = very bad, 10 = very good) symptoms on a visual analog scale. We used logins/access days as a metric of engagement as it provided a measure of “stickiness.”

#### The Transition Readiness Assessment Questionnaire (TRAQ-5)

The TRAQ-5 contains 20 questions that measure the self-management skills needed for the transition to adult care in five domains: managing medication, appointment keeping, tracking health issues, talking with providers, and managing daily activities [29]. Items are rated on a 5-point Likert scale of 1 = “No, I do not know how” to 5 = “Yes, I always do this when I need to.” The overall score was used in analyses and was calculated by averaging the scores of answered items. The TRAQ-5 has been used previously in AYA with SCD [30]. The Cronbach’s alpha in the present study was .93, which indicates excellent internal consistency.

#### Patient Activation Measure (PAM-13)

The PAM-13 contains 13 statements that measure self-efficacy skills, including perceived knowledge, skill, and confidence to manage one’s health and health care. The PAM-13 has been used extensively in other chronic-illness populations [31]. Items are rated on a 4-point Likert scale of 1 = “Disagree Strongly” to 4 = “Strongly Agree.” Raw scores range from 13 to 52 and are converted to scores that range from 0 to 100. The overall score was used in analyses and was calculated by averaging the scores of answered items. The PAM-13 has been used previously in AYA with SCD [12]. The Cronbach’s alpha in the present study was .87, which indicates excellent internal consistency.

### Data analysis plan

All analyses were conducted using the R statistical package [32]. Descriptive and summary statistics were utilized to report AYA characteristics, mobile app usage, self-management goals, and pain and mood ratings. Pearson correlations assessed relationships between continuous variables. Because of multicollinearity between variables, separate hierarchical linear regressions were conducted to determine whether randomization blocking variables (e.g., age group or disease severity) predicted engagement with the mobile app (i.e., logins and completion of self-management goals) using the lm function. Additionally, two separate hierarchical linear regressions were conducted to determine whether engagement with the mobile app predicted post-treatment self-management skills and self-efficacy. In both analyses, we controlled for baseline scores to determine the portion of the post-treatment scores not explained by baseline scores.

Holm adjustments controlled for Type 1 error. Effect sizes (partial eta-squared; *η*^2^) were calculated for all effects. *η*^2^ = .01, .06, and .14 represented small, medium, and large effect sizes, respectively. We used bias-corrected and accelerated 95% confidence intervals (CI) as they adjust for possible bias and determined significance at an alpha level of *p* < .05 two-tailed.

## Results

### Participants

The sample included AYA who received the intervention and were aged 13–21 years (*M*_age_ = 16.7 years; 54% female; 46% HbSS genotype; all African American/Black).

### iManage for SCD App Usage

AYA with SCD had access to the adjunct mobile app during the intervention for an average of 86 days (*SD* = 33.41, range = 48 – 185). During this period, AYA logged into the mobile app a total of 519 times for an average of 20 logins per participant (*SD* = 13.16, range = 2 – 48). Therefore, on average, AYA engaged in the mobile app about once every 8 days (*SD* = 9.1, range = 1 – 45). There was considerable variability in the engagement of our AYA cohort. For example, 5 participants (19%) logged into the mobile app on average every 1 – 2 days, while 4 participants (15%) logged into the app about once every 12 days.

### Self-management Goals

AYA with SCD viewed the self-management goal page of the mobile app a total of 397 times for an average of once every 11 days (*SD* = 13.0, range = 4 – 57). All AYA created self-management goals (*M* = 5.7, *SD* = .72, range = 4 – 7), but only 54% of AYA tracked completion within the app. Of the 149 self-management goals created by the entire sample during the intervention, only 37 (25%) were recorded as completed on the app. Themes of self-management goals varied, but increasing exercise, completing schoolwork, drinking more water, and making changes to sleep routine were the most commonly identified goals (see Table 1).

**Table 1 |:** Themes of self-management goals recorded on the mobile app

Type of Goal	Example	Percentage of Total Goals Recorded	Percentage of Goals Completed
Exercise	“I will exercise every day for the next two weeks”	18%	12%
Schoolwork	“Finish my make-up homework by Thursday”	14%	14%
Drink more water	“Drink 3 bottles of water every day between now and next Tuesday”	11%	23%
Change sleep schedule	“Go to bed by 11 on Wednesday and Monday night”	11%	25%
Eat more healthfully	“Eat more vegetables for dinner on Thursday”	7%	27%
Plan for the future	“Take the driving test Thursday early in the morning”	9%	15%
Change habits	“Turn off my phone for 2 hours Monday – Friday”	6%	88%
CBT techniques	“Do guided imagery at least one night before bed”	5%	12%
Family/friends	“Get costume and be Santa Claus for daughter and nieces”	5%	50%
Chores	“Make my bed four days this week: Wednesday, Thursday, Saturday & Sunday”	5%	29%
Food	“Cook my famous stuffing for Thanksgiving, Thursday morning”	5%	14%
Medication Adherence	“Take medicine every day at night”	4%	20%

*Note.* CBT = Cognitive Behavioral Therapy

**Figure 3 |:**
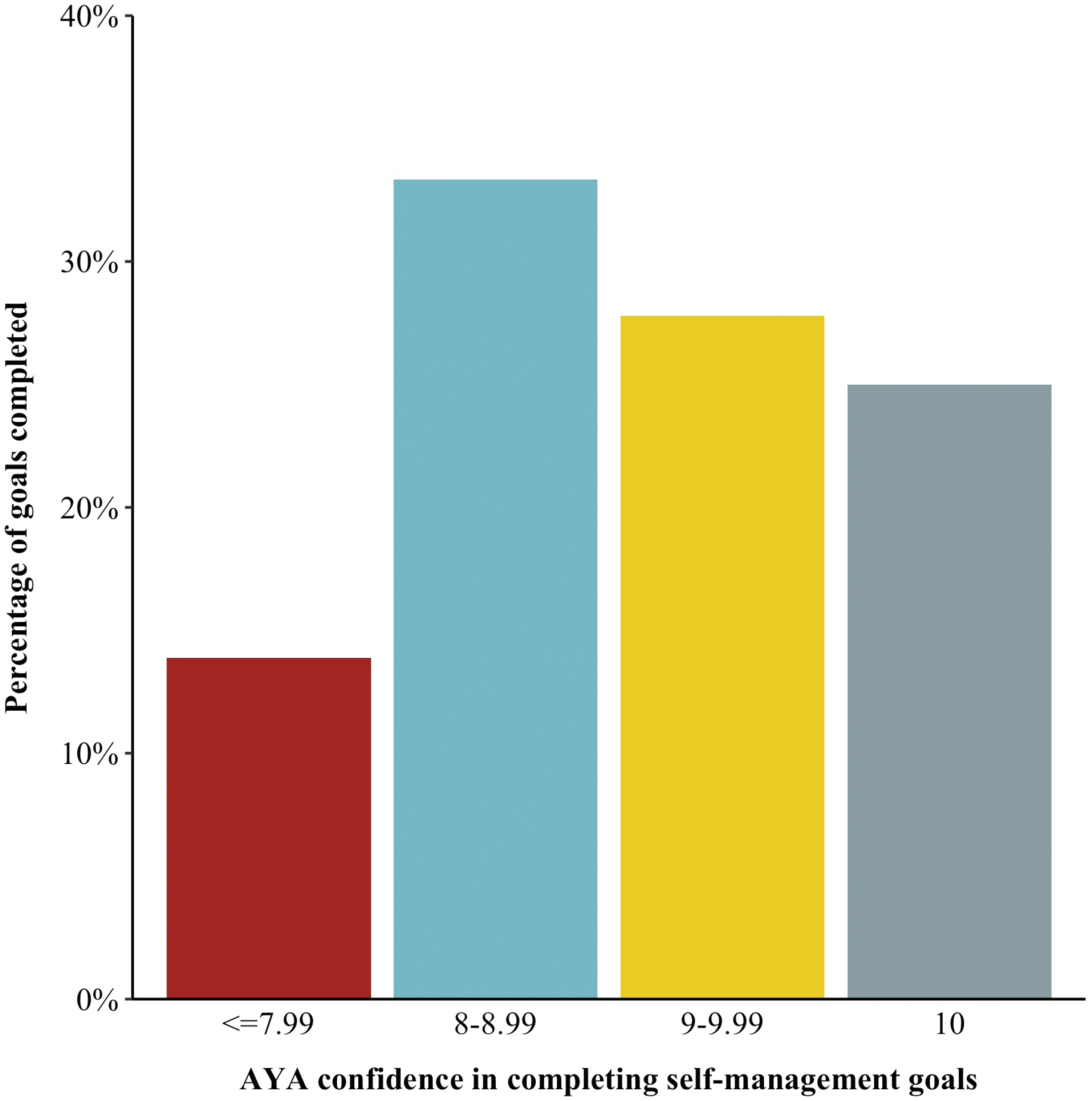
The percentage of self-management goals completed by AYA with SCD in relation to their confidence when goals were created on the iManage for SCD app

Confidence of AYA with SCD that they would complete their self-management goals was generally high (*M* = 8.5, *SD* = 1.0, range = 7 – 10). AYA with a confidence level of 7 were least likely to complete their self-management goals (14%), but AYA with a confidence level of 8 were most likely to complete their self-management goals (33%) (see Figure 3).

### Pain and Mood

Twenty-four AYA with SCD (92% of the sample) completed 383 pain diaries on the mobile app during the intervention at a rate of once every 11 days (*SD* = 13.0, range = 0 – 127). Two AYA did not complete a pain diary. Only 18 AYA (69% of the sample) rated their pain symptoms with an average of 9 ratings per participant (*SD* = 10.31, range = 1 – 40). Pain ratings were of mild severity (*M* = 4.9, *SD* = 1.8, range = 0 – 7). Most AYA did not report having unplanned hospital contacts or hospitalizations. The most commonly used strategies for relieving pain were resting and drinking water (see Table 2). Twenty-five AYA (96% of the sample) rated their mood symptoms with an average of 26 ratings per participant (*SD* = 18.9, range = 1 – 55). Mood ratings were generally high (*M* = 7.2, *SD* = 1.6, range = 4 – 10).

**Table 2 |:** Pain diary entries for adolescents and young adults with sickle cell disease

Pain Diary Questions	Number of times response recorded	Number of AYA who responded n (%)
*Because of my Sickle Cell Disease…*		
Unplanned (emergency, sudden) doctor’s office or clinic visit	4	4 (15.38)
I went to the emergency room	1	1 (3.85)
I was in hospital overnight	9	3 (11.54)
*What steps did you take to relieve your pain today?*		
Tell a Parent/Adult	24	10 (38.46)
Rest	98	17 (65.38)
Water	85	17 (65.38)
Distraction Techniques	37	8 (30.77)
Warm Heating Pad	37	11 (42.31)
Contacted Sickle Cell Disease Clinic	5	4 (15.38)
*What medications have you taken today, and how much?*		
Acetaminophen	28	5 (19.23)
Ibuprofen	20	7 (26.92)
Oxycodone	21	8 (30.77)
Oxycodone Hydrochloride	14	4 (15.38)

Note. AYA = adolescents and young adults

### iManage for SCD

#### Group Differences

Separate hierarchical linear regression analyses demonstrated that AYA in the older age group (18 – 21 year-olds), *F*(1, 24) = 3.72, *r*^2^ = .10, 95% CI[.00, .37], *p* = .07, logged on to the mobile app less frequently, although this finding was not statistically significant. Greater disease severity (i.e., 3 or more pain episodes in the past 3 years and a previous history or stroke or acute chest syndrome) did not predict mobile app logins, *r*^2^ = .05, *p* = .14. However, older AYA, *F*(1, 24) = 6.53, *r*^2^ = .18, 95% CI[.01, .45], *p* = .02, and those with greater disease severity, *F*(1, 24) = 6.19, *r*^2^ = .17, 95% CI[.00, .44], *p* = .02, completed fewer self-management goals.

#### Self-management Outcomes

Significant correlations were demonstrated between engagement (i.e., more logins) with the mobile app and higher mood ratings (*r* = .54, 95% CI[.18, .77], *p* = .006) and lower pain ratings (*r* = -.48, 95% CI[-.77, -.02], *p* = .04). Although the relationship was not statistically significant, logging into the mobile app more frequently was associated with completing more self-management goals with a small-to-moderate effect size (*r* = .35, 95% CI[-.05, .65], *p* = .08).

Hierarchical linear regression analyses (see Table 3) demonstrated that after controlling for scores at baseline, the overall model of the effect of engagement with the mobile app and self-management skills was significant *F*(3, 22) = 3.35, *p* = .04. Specifically, the number of logins to the mobile app significantly predicted AYA-reported self-management skills (TRAQ-5, *b* = 0.04, *p* = .05, 95% CI = 0.00, 0.07), with a non-significant trend observed for self-efficacy (PAM-13) (*p* > .05) (see Table 3).

**Table 3 |:** Regression models using self-management skills (TRAQ-5) and self-efficacy (PAM-13) as the dependent variables and logging into the mobile app and completing self-management goals (i.e., engagement) as predictors

Effect	*B*	*B* 95% CI [LL, UL]	*β*	*SE*	*p*	*Partial* η^2^	Δ R^2^	Fit
**Self-management skills** **(TRAQ-5)**								
(Intercept)	2.91	[1.56, 4.27]						
Baseline TRAQ-5 scores	0.37	[0.01, 0.74]	.38	.18	.05*	.17	–	
Logging into mobile app	0.04	[0.00, 0.07]	.40	.02	.05*	.17	.08	
Completed self-management goals	-0.16	[-0.35, 0.02]	-.35	.09	.08	.14	.11^†^	
								*R* ^*2*^ = .313*
								95% CI[.00, .50]
**Self-efficacy (PAM-13)**								
(Intercept)	61.24	[31.66, 90.82]						
Baseline PAM-13 scores	0.28	[-0.10, 0.66]	.29	.18	.14	.09	–	
Logging into mobile app	0.60	[-0.08, 1.28]	.36	.33	.08	.13	.15*	
Completed self-management goals	0.71	[-2.91, 4.34]	.08	1.75	.68	.01	.01	
								*R* ^*2*^ = .249
								95% CI[.00, .44]

*Note. B* represents unstandardized regression weights. *LL* and *UL* indicate the lower and upper limits of a confidence interval, respectively. *β* = standardized regression weights; *SE* = Standard Error; *Partial* η ^2^ = partial eta squared; * = *p* < .05, ^†^ = *p <* .10

## Discussion

Results from this preliminary study suggest that a mHealth app was successfully integrated into an RCT for AYA with SCD and resulted in a clinically meaningful change in self-management. A co-design process was used in the development of the app [26] as it is critical to include AYA input early in the development process to promote engagement [33,34]. AYA with SCD who had more engagement with the mobile app experienced less pain and better mood symptoms. When experiencing pain, AYA most often rested or drank water to relieve their pain, but reported using medications infrequently. Regarding intervention primary outcomes, logging on to the mobile app predicted improved self-management skills and self-efficacy with clinically meaningful effects. Completion of self-management goals did not predict self-efficacy.

Surprisingly, completion of goals predicted worse self-management skills, which was in the opposite direction to our hypothesis (*p* = .08). It remains unclear why completing more self-management goals on the mobile app would result in fewer AYA-reported self-management skills. One plausible explanation is that even those AYA who completed self-management goals were unsuccessful at completing more complex self-management tasks (e.g., scheduling appointments). Thus, when they considered their ability to track health issues and manage their daily activities, their inability to complete complex tasks overshadowed success with smaller tasks. Further, engaging with the app may also have led AYA to re-evaluate their skills levels. AYA could also see their peers’ goals, so they may have made comparisons resulting in lower levels of reported self-management. However, these hypotheses require further research.

Despite AYA with SCD reporting the acceptability and utility of the mobile app [23] and reporting that tracking their pain, mood, and completing their self-management goals was beneficial [26], overall engagement during the intervention was variable and was limited for some AYA. Some AYA engaged with the app infrequently and did not create or complete self-management goals; others could be defined as “super users” who logged into the app almost daily, frequently monitored their pain, and created self-management goals. It is possible that some AYA completed self-management goals, but did not report them on the mobile app. However, analyses to elucidate the reasons for variable levels of engagement demonstrated that younger AYA and those with less disease severity logged into the app more frequently and were most successful at completing self-management goals, particularly related to drinking more water, making changes to their sleep schedule, or making healthier dietary choices. Increased confidence also improved the likelihood that AYA completed their self-management goals, but the relationship was not linear. The largest percentage of goals completed were the goals where AYA reported confidence of 8 out of 10. Although it is conceivable that AYA whose self-management and self-efficacy improved during the intervention became more engaged [35], our data suggest that AYA engagement is key.

AYA provided detailed qualitative feedback about the intervention [26]. In relation to the mobile app, they used the text message features infrequently and they reported that they would have preferred if they could have access on their own device. Given this feedback, modifications to increase engagement in future interventions may include adapting the frequency of app reminder notifications (e.g., fewer logins generates more notifications). Moreover, although the intervention was designed to be developmentally appropriate, we found older AYA were less engaged than the younger cohort. This finding suggests that additional tailoring may be needed (e.g., older AYA receive different text messages). Finally, making the app easily available for download on their own phone or tablet may increase engagement. Although our research team encouraged peer interactions, communication through the mobile app was limited. Finding ways to incorporate social networking may increase active engagement and allow AYA to share their experiences and offer each other emotional and informational support. These peer-to-peer interactions could be facilitated through video, moderated chatrooms, or through peer-developed creative content; however, risks to patient privacy must be considered when testing these features. Future research would also have to consider the time, resources, monitoring, and maintenance needed to implement these features sustainably.

Previous research has shown that increasing caregiver involvement improves engagement in digital interventions [36]. Given that older AYA had the most difficulty with engagement in the present study, allowing them to designate a person other than a caregiver (i.e., older sibling, friend) to increase accountability may aid independence from their caregiver/s. For all features, the app research team needs to consider the executive function challenges faced by AYA with SCD [37] and look to incorporate immediate feedback and have tasks broken into small steps. Other features could allow AYA to assess their rate of progress during goals, provide a review of completed goals that include an assessment of what worked and why, and include discussions of approaches that might be modified to be more successful in the future.

There are limitations to the present study. Our sample size was small and although AYA were randomized to the treatment arm, it was difficult to detect smaller effects and possible interactions between variables (e.g., age and disease severity) could not be assessed. The intervention took place over 6 weeks, with an 8-week booster session. Assessments of primary outcomes at 6 or 12 months would provide additional information about whether gains in self-management were sustained. Our study also took place outside of regular clinic visits. It is difficult to know the role of incentives with engagement and if all or just some components the intervention are needed to maintain effectiveness. Therefore, more research is needed to determine how to optimally integrate mHealth interventions within the larger healthcare system. We also used logins/access days as a metric of engagement, instead of time spent in the app, as user sessions can be challenging to accurately define on mobile platforms. However, future research might consider using a platform that counts a user session from when they open and interact with the app until 30 minutes of inactivity (even though this metric might still overestimate engagement). Further, this study was conducted at a single SCD center in the midwest of the United States and the cohort may not be representative of the AYA with SCD population in the rest of the United States or other countries. An important consideration for future mHealth interventions that include AYA with SCD would be the inclusion of economic data to assess cost-effectiveness to understand the impact of mHealth on self-management [38]. Additionally, in light of changes related to the COVID-19 pandemic, it will be critical to understand the benefits and key components of mHealth approaches to optimize telehealth for AYA with SCD.

Our mobile app seems to be a feasible way to engender behavior change in AYA with SCD. We used a co-design in the development process, utilized a behavioral conceptual framework, incorporated the app as an adjunct for an RCT, and had AYA create and track their self-management goals. Few previous studies of AYA with SCD have included all of these components [8]. Similar to other studies, we had features to increase engagement including recording of mood and symptoms, calendar tracking, and the ability to communicate with peers. Engagement with the app was similar or exceeded other mHealth studies of AYA with SCD [8].

Our preliminary study provides support for the mobile app to improve self-management and self-efficacy among AYA with SCD. Lessons learned from our study indicate that it can be challenging to maintain engagement in mHealth for AYA with SCD, but for those who do engage there are clinically meaningful benefits related to self-management goals, documentation of pain symptoms, and mood. Thus, our findings indicate that an adjunct mHealth app can be effectively integrated into a clinical trial and is related to positive outcomes. Although there are challenges to address, mHealth has the potential to bring about changes in behavior and improve health in AYA with SCD.

## Funding

The study reported was supported in part by Eunice Kennedy Shriver National Institute of Child Health & Human Development of the National Institutes of Health under Award Number R21HD084810 (LEC). Anna Hood was supported in part by a grant from the National Heart, Lung, and Blood Institute (1F32HL143915).

## COMPLIANCE WITH ETHICAL STANDARDS

**Authors’ Statement of Conflict of Interest and Adherence to Ethical Standards:** All authors declare that they have no conflicts of interest.

**Human Rights:** All procedures performed in studies involving human participants were in accordance with the ethical standards of the institutional and/or national research committee and with the 1964 Helsinki declaration and its later amendments or comparable ethical standards. The Institutional Review Board of the medical center approved the study.

**Informed Consent:** Informed consent was obtained from all individual participants included in the study.

**Welfare of Animals:** This article does not contain any studies with animals performed by any of the authors.

**Data availability:** This study and our analysis plan were not formally pre-registered. Materials used to conduct the study are not publically available. De-identified data and analytic code used to conduct the analyses presented in this study are not available in a public archive. They may be available by emailing the corresponding author (as allowable according to institutional IRB standards).
